# Prevalence of Dyslipidemia in Urban and Rural Areas of the Northwest of Iran: The Sociodemographic, Dietary and Psychological Determinants

**Published:** 2019-05

**Authors:** Jafar Sadegh TABRIZI, Leila NIKNIAZ, Homayoun SADEGHI-BAZARGANI, Mostafa FARAHBAKHSH, Zeinab NIKNIAZ, Mahdieh ABBASALIZAD FARHANGI, Elham EGHBALI

**Affiliations:** 1. Tabriz Health Services Management Research Center, Faculty of Management and Medical Informatics, Tabriz University of Medical Sciences, Tabriz, Iran; 2. Tabriz Health Services Management Research Center, Tabriz University of Medical Sciences, Tabriz, Iran; 3. Department of Statistics and Epidemiology, Road Traffic Injury Research Center, Tabriz University of Medical Sciences, Tabriz, Iran; 4. Research Center of Psychiatry and Behavioral Sciences, Tabriz University of Medical Sciences, Tabriz, Iran; 5. Liver and Gastrointestinal Diseases Research Center, Tabriz University of Medical Sciences, Tabriz, Iran; 6. Drug applied Research Center, Tabriz University of Medical Sciences, Tabriz, Iran; 7. Liver and Gastrointestinal Diseases Research Center, Tabriz University of Medical Sciences, Tabriz, Iran

**Keywords:** Dietary intake, Dyslipidemia, Iran, Smoking, Anxiety

## Abstract

**Background:**

As dyslipidemia is a preventable risk factor for Coronary heart disease (CHD), precise estimation of its prevalence and determinants is crucial for proper development of health actions. This population-based study aimed at investigating the socioeconomic, dietary and psychological determinants of dyslipidemia in Iran.

**Methods:**

The data (n=700) for this study were collected in 2015 as a part of the major Lifestyle Promotion Project (LPP) conducted in East Azerbaijan (urban and regional parts). The data for socio-demographic status, dietary information, and physical activity and anxiety levels were collected through validated questionnaires. Then, physical examinations including blood pressure, body mass index (BMI) and conicity index were performed. The levels of serum lipids were measured by enzymatic colorimetric methods.

**Results:**

The prevalence of hypercholesterolemia, high LDL-C, hypertriglyceridemia, low HDL-C and dyslipidemia was 29.4%, 10.3%, 62.3%, 41.4%, 83.3% respectively. The mean TC (184.3±41.2 vs. 174.5±38.1 mg/dl), LDL-C (94.6±30.3 vs. 88.1±28.7 mg/dl) and HDL-C (46.7±10.4 vs. 39.5±8.0 mg/dl) in women were significantly higher than men (*P*<0.05). However, the mean of TG (182.3±119.3 vs. 145.1±87.8 mg/dl) was significantly higher in men compared to women (*P*<0.05). Obesity, family history of dyslipidemia, sedentary lifestyle, smoking habits, salt intake, and anxiety were risk factors for different components of dyslipidemia in men and women.

**Conclusion:**

Dyslipidemia is a major health problem in northwest of Iran. Focusing on screening, regular drug intake, proper nutrition, physical activity, and changing lifestyles of patients with dyslipidemia are essential.

## Introduction

Cardiovascular disease (CVD) considered as one of the main health problems in Iran (1). A major modifiable factor for the development of CVD is dyslipidemia (2). The prevalence of dyslipidemia is increasing increases constantly as a result of adverse lifestyle changes (3).

In an earlier study in Iran, the prevalence of hypercholesterolemia and hypertriglyceridemia estimated to be 27.1% and 19.4% respectively among Iranian adults (4). In Iran, different risk factors including changing in social and economic status of population, huge lifestyle changes, such as Westernization of diet, reduced physical activity and long-term sedentary work, all of considered as main contributors of increase in prevalence of dyslipidemia (5).

As dyslipidemia is a preventable risk factor for CVD, exact estimation of its prevalence and patterns is crucial for development of appropriate programs for controlling and managing of harmful clinical consequences.

This paper presents the first phase of a comprehensive community-based intervention program for prevention and control of NCDs and their risk factors in Iran (LPP study). To the best of our knowledge, no recent study has been conducted to report the prevalence of dyslipidemia on population level in Iran.

To address this gap in knowledge, this study intends to generate relevant information that helps to understand the patterns of dyslipidemia in populations where the prevalence of non-communicable diseases (NCDs) risk factors is growing rapidly.

## Methods

The Lifestyle Promotion Project (LPP) dataset was used in the present study. LPP was a cross-sectional population based study conducted in East Azerbaijan (urban and regional parts)-Iran. The exact method of sampling is described in detail elsewhere (6). Five participants were enrolled in each 150 selected clusters (750 participants). By excluding incomplete information, the data of 700 men and women (age range: 15–65 yr) were analyzed. The information of socio-demographic characteristics, physical activity status, severity of general anxiety, dietary data, anthropometric measures, and blood pressure were collected (6).

The Ethics Committee of Tabriz University of Medical Science (registration number: 1394.383) was approved the study and informed consent was obtained from all participants.

About 5 ml blood sample was collected after an overnight fast. The samples were immediately centrifuged and the serum samples were separated. Enzymatic colorimetric method (by commercial kit of Pars Azmone, Tehran, Iran) was used for meaning the serum level of total cholesterol, High-density lipoprotein and triglyceride and LDL-C was calculated by Friedewald equation (7). We used the NCEP cutoffs for borderline hypercholesterolemia (≥200 mg/dl), hypertriglyceridemia (≥150 mg/dl), high levels of LDL-C (≥ 130 mg/dl) and low levels of HDL-C (<40 mg/dl in males, <50 mg/dl in females).

All statistical analyses were performed in SPPS (ver. 18, Chicago, IL, USA). Continuous and categorical variables were reported as means and standard deviations (SDs) and proportions respectively. Independent t-test and chi-square were used for between-group comparisons. The multinomial logistic regression models were used to examine the relationships between dyslipidemia components and associated factors by adjusting for covariates including residency area, education, employment, smoking, physical activity, age, anxiety, family history of dyslipidemia and dietary factors. A significance level of 0.05 was used.

## Results

Table 1 presents the general characteristics of participants stratified by residency area. About 52.5% of participants were living in urban areas and 47.4% of them were living in regional areas.

**Table 1:** General characteristics of participants in urban and regional areas

***Characteristics***	***Urban (n= 368)***	***regional (n= 332)***	***Total (n= 700)***
Age (yr), % (n)^*^			
18–25	4.3 (16)	5.2 (17)	4.8 (33)
26–35	14.7 (54)	15.3 (51)	15.0 (105)
36–45	26.3 (97)	27.0 (90)	26.6 (187)
46–55	27.2 (100)	31.5 (104)	29.2 (204)
56–65	27.6 (101)	21.0 (70)	24.4 (171)
Marital status, % (n)			
Married^*^	90.7 (334)	91.3 (303)	91.0 (637)
Occupational status, % (n)			
Employed or self employed	32.7 (120)	35.7 (118)	34.2 (238)
Student	3.3 (12)	2.1 (7)	2.7 (19)
Unemployed	64.0 (236)	62.2 (207)	63.2 (443)
Educational status, % (n)^*^			
Illiterate	8.9 (33)	18.3 (61)	13.4 (94)
Under graduate	73.2 (269)	69.2 (230)	71.3 (499)
University	17.9 (66)	12.5 (41)	15.1 (107)
Smoking habit, % (n)^*^			
yes	6.4 (23)	8.8 (29)	7.4 (52)
Occasionally	2.1 (8)	14.7 (49)	8.1 (57)
No	91.5 (337)	76.5 (254)	84.4 (591)
Physical activity, % (n)^*^			
Inactive	51.6 (190)	21.3 (71)	37.3 (261)
Minimally active	31.3 (115)	28.1 (93)	29.7 (208)
Health enhancing activity	17.1 (63)	50.6 (168)	34.3 (231)

(*P*<0.05), differences tested by chi-square test

Prevalence of borderline and high serum lipids in urban and regional areas is shown in Table 2. The residents of regional areas significantly had lower HDL-C level and higher dyslipidemia compared with urban ones.

**Table 2:** Prevalence of borderline and high serum lipids in urban and regional areas

***Lipid profile***	***Urban (n= 368)***	***regional (n= 332)***	***Total (n= 700)***
Prevalence of borderline high and high TC, % (n)	29.9 (110)	29.2 (97)	29.6 (207)
Prevalence of borderline high and high LDL-C, % (n)	10.1 (37)	10.6 (35)	10.3 (72)
Prevalence of borderline high and high TG, % (n)	43.2 (159)	40.4(134)	41.8 (293)
Prevalence of low HDL-C, % (n)^*^	57.1 (210)	67.5 (224)	62.0 (434)
Prevalence of dyslipidemia, % (n)^*^	80.5 (296)	86.5 (287)	83.3 (583)

(*P*<0.05), differences tested by chi-square test

The prevalence of dyslipidemia, hypertriglyceridemia, hypercholesterolemia, hyper LDL-cholesterolemia, and hypo HDL-cholesterolemia was 83.3%, 62.3%, 29.4%, 10.3%, and 41.4% respectively. Moreover, 8.6% of Iranian adults had mixed dyslipidemia (hyper LDL-cholesterolemia with either hypo HDL-cholesterolemia and/or hypertriglyceridemia), with nearly 2.8% had all three lipid abnormalities (it has not been shown).

The prevalence of different lipid abnormalities across different age and sex groups is presented in Table 3. The mean total cholesterol (184.3±41.2 vs. 174.5±38.1 mg/dl), LDL-C (94.6±30.3 vs. 88.1±28.7 mg/dl) and HDL-C (46.7±10.4 vs. 39.5±8.0 mg/dl) in women were significantly higher than men. However, the mean of TG (182.3±119.3 vs. 145.1±87.8 mg/dl) was significantly higher in men compared to women. Middle-aged participants had significantly higher prevalence of dyslipidemia in compared with younger participants.

**Table 3:** The prevalence of high total cholesterol, high LDL-C, and high TG, low HDL-C and dyslipidemia by age and sex

***Variables***	***Men***	***Women***	***P value^†^***
Prevalence of borderline high and high TC, (%)	22.3	34.0	0.003
15–25 (yr)	7.7	16.7	0.399
26–35	11.5	21.3	0.30
36–45	19.2	24.7	0.46
46–55	26.4	34.4	0.32
56–65	28.3	56.5	0.002
*P*-value ^‡^	0.23	<0.001	
Prevalence of borderline high and high LDL-C, (%)	8.7	11.5	0.287
15–25 (yr)	0.0	8.3	0.14
26–35	3.8	4.3	0.91
36–45	5.8	11.3	0.28
46–55	3.8	9.5	0.20
56–65	19.2	17.4	0.79
*P*-value ^‡^	0.017	0.26	
Prevalence of borderline high and high TG, (%)	51.6	34.1	<0.001
15–25 (yr)	30.8	8.3	0.039
26–35	26.9	27.7	0.94
36–45	57.7	31.3	0.003
46–55	62.3	30.2	<0.001
56–65	53.8	49.3	0.62
P-value ^‡^	0.02	0.14	
Prevalence of low HDL-C, (%)	55.9	67.0	0.009
15–25 (yr)	53.8	66.7	0.522
26–35	69.2	66.0	0.77
36–45	63.5	76.5	0.1
46–55	54.7	63.5	0.29
56–65	47.2	60.9	0.13
*P*-value ^‡^	0.32	0.28	
Prevalence of dyslipidemia, %	80.8	85.0	0.2
15–25 (yr)	69.2	90.9	0.041
26–35	76.9	78.7	0.86
36–45	90.2	90.1	0.98
46–55	84.9	81.3	0.57
56–65	73.6	88.4	0.03
*P*-value ^‡^	0.15	0.27	
Prevalence of dyslipidemia awareness, %	3.3	5.9	<0.001
15–25 (yr)	0.2	0.0	0.37
26–35	0.0	0.1	0.26
36–45	2.0	2.8	0.30
46–55	6.7	8.0	0.33
56–65	5.9	18.8	<0.001
*P*-value^‡^	<0.001	<0.001	

† Differences tested by chi-square test

‡ Differences tested by Kruskal-Wallis

Furthermore, 9.2% of the dyslipidemic patients were aware of their disease, however, only 55.0% of the aware ones were receiving medication or TLC.

The association between borderline high and high serum lipids with demographic, socio-economic and lifestyle factors were presented in Tables 4 and 5.

**Table 4:** Logistic regression analysis for the association of borderline high and high serum lipids and demographic, socio-economic, and lifestyle factors in men

***Variables***	***Borderline high and high LDL-C Adjusted OR (95% CI)***	***Borderline high and high TC Adjusted OR (95% CI)***	***Borderline high and high TG Adjusted OR (95% CI)***	***Low HDL-C Adjusted OR (95% CI)***
Age groups(yr0				
15–25	1.00	1.00	1.00	1.00
26–35	8.70 (0.02,17.6)	1.39 (0.08, 3.37)	1.01 (0.18, 2.15)	2.53 (0.72, 5.39)
36–45	2.96 (0.40, 5.74)	1.93 (0.18, 2.71)	1.08 (0.09,1.74)	1.46(0.16, 3.13)
46–55	1.91 (0.32,3.16)	2.14 (0.91, 7.76)	1.09 (1.00, 3.19)	1.82 (0.38, 4.32)
56–65	1.00 (0.13, 1.85)	2.05 (0.59, 3.30)	1.19 (0.44, 2.09)	1.78 (0.18, 3.77)
Residential place				
Urban	1.00	1.00	1.00	1.00
Rural	0.28 (0.06, 1.94)	0.16 (0.01, 2.5)	0.33 (0.03, 3.10)	4.76 (1.46, 15.83)^*^
Marital status				
Single	1.00	1.00	1.00	1.00
Married	0.87 (0.05, 2.85)	5.51 (0.06, 2.93)	0.29 (0.01, 7.60) ^*^	2.18 (0.18, 3.63)
Occupational status				
Employed	1.00	1.00	1.00	1.00
Student	0.81 (0.06, 1.72)	1.15 (0.29, 4.63)	1.08 (0.33, 2.94)	1.14 (0.46, 2.15)
Unemployed	0.79 (0.07, 8.25)	1.06 (0.02, 1.19)	7.80 (0.47, 25.65)	2.21 (0.17, 5.73)
Educational status				
Illiterate	1.00	1.00	1.00	1.00
Under graduate	1.55 (0.31, 7.73)	0.01 (0.00, 3.66)	0.23 (0.03, 1.55)	1.47 (0.04, 4.91)
College	1.33 (0.11, 15.13)	0.06 (0. 01, 1.95)	0.10 (0.01, 3.62)	1.27 (0.03,4.42)
Smoking habit				
yes	1.00	1.00	1.00	1.00
Occasionally	2.93 (0.11, 10.14)	0.34 (0.17, 1.09)	1.17 (0.34,1.99)	0.12 (0.01, 1.19)
No	0.72 (0.05, 2.56)	0.22 (0.09, 0.75) ^*^	0.15 (0.07, 1.57)	0.24 (0.03, 0.84) ^*^
Physical activity				
Inactive	1.00	1.00	1.00	1.00
Minimally active	0.78 (0.17, 3.81)	0.48 (0.03, 2.67)	0.34 (0.04, 2.44)	0.44 (0.11, 1.49)
Health enhancing activity	0.42 (0.09, 2.37)	0.54 (0.38, 1.71)	0.40 (0.12, 1.38)	0.62 (0.18, 1.76)
Family history of dyslipidemia				
No	1.00	1.00	1.00	1.00
Yes	1.56 (0.32, 7.56)	13.33 (5.33, 22.81) ^**^	1.37 (0.07, 3.68)	1.16 (0.12, 3.82)
Overweight and obesity				
No	1.00		1.00	
Yes	2.99 (1.72, 8.98) ^**^	24.56 (1.81, 33.34) ^*^	14.66 (2.03, 105.10) ^***^	5.95 (1.11, 31.50) ^*^
High conicity index				
No	1.00	1.00	1.00	
Yes	1.49 (0.11, 5.61)	3.29 (0.78, 4.71)	1.58 (1.15, 4.16) ^*^	1.86 (0.11, 2.95)
Generalized Anxiety Disorder				
No anxiety	1.00	1.00	1.00	1.00
Mild	1.57 (0.17, 1.88)	1.58 (0.51, 6.60)	1.32 (0.44, 3.96)	0.75 (0.12, 1.09)
Moderate	1.87 (0.14, 2.01)	1.87 (0.70, 2.95)	2.67 (0.97, 4.82)	1.16 (0.44, 1.88)
Severe	1.65 (0.04, 1.91)	2.05 (0.93, 6.73)	5.94 (1.70, 42.29) ^*^	2.37 (0.30, 6.61)
Having hypertension				
No	1.00	1.00	1.00	
Yes	1.15 (0.31, 4.39)	1.54 (0.04, 6.40)	1.51 (0.65, 3.71)	1.82 (0.38, 4.29)
Having diabetes				
No	1.00	1.00	1.00	
Yes	1.76 (0.83, 6.74)	1.09 (0.11, 3.22)	2.30 (0.86, 7.14)	4.20 (0.93, 8.69)
Salt intake (gr/day)				
Tertile 1	1.00	1.00	1.00	1.00
Tertile 2	1.24 (0.71, 3.51)	51.32 (2.71, 969.8) ^***^	1.62 (0.75, 2.96)	1.37 (0.71, 3.08)
Tertile 3	1.12 (0.30, 3.29)	57.69 (1.40, 238.2) ^**^	1.29 (0.43, 3.00)	1.18 (0.26, 2.19)
Refined carbohydrate intake (gr/day)				
Tertile 1	1.00	1.00	1.00	1.00
Tertile 2	1.08 (0.03, 2.85)	1.55 (0.83, 3.49)	1.14 (1.02, 5.19) ^*^	0.9 (0.09, 2.33)
Tertile 3	1.09 (0.10, 1.96)	1.47 (0.32, 3.36)	1.21 (1.06, 5.63) ^*^	1.03 (0.01, 1.26)
Saturated fat intake (gr/day)				
Tertile 1	1.00	1.00	1.00	1.00
Tertile 2	1.11 (0. 23, 2.36)	1.46 (0.47, 4.52)	1.97 (0.15, 3.86)	1.75 (0.70, 3.33)
Tertile 3	1.19 (0.73, 2.97)	1.93 (0.84, 5.34)	1.15 (0.20, 3.19)	1.88 (0.73, 3.20)

* *P*<0.05,

** *P*<0.01,

*** *P*<0.001, Multiple logistic regressions considering the simultaneous effect of all the explanatory variables

**Table 5:** Logistic regression analysis for the association of borderline high and high serum lipids and demographic, socio-economic, and lifestyle factors in women

***Variables***	***Borderline high and high LDL-C Adjusted OR (95% CI)***	***Borderline high and high TC Adjusted OR (95% CI)***	***Borderline high and high TG Adjusted OR (95% CI)***	***Low HDL-C Adjusted OR (95% CI)***
Age groups(yr)				
15–25	1.00	1.00	1.00	1.00
26–35	1.55 (0.03,8.19)	1.34 (0.90, 3.12)	1.28 (0.06, 2.44)	1.70 (0.19, 4.29)
36–45	1.20 (0.14, 3.18)	1.70 (0.97, 5.55)	3.57 (0.22,6.51)	1.51 (0.19, 3.82)
46–55	1.30 (0.16,6.38)	1.30 (0.18, 3.36)	3.03 (0.18, 5.14)	1.24 (0.84, 3.79)
56–65	1.57 (0. 42, 2.22)	2.04 (0.24, 3.30)	4.93 (0.28,9.38)	1.40 (0.46, 3.51)
Residential place				
Urban	1.00	1.00	1.00	1.00
Rural	0.78 (0.0.23, 2.42)	1.20 (0.51, 2.71)	1.70 (0.69, 2.09)	1.74 (1.08, 4.84) ^*^
Marital status				
Single	1.00	1.00	1.00	1.00
Married	2.25 (0.16 3.14)	0.41 (0.06, 2.20)	0.43 (0.06, 3.22)	1.41 (0.24, 4.08)
Occupational status				
Employed	1.00	1.00	1.00	1.00
Student	0.41 (0.01, 2.63)	0.59 (0.22, 1.29)	0.31 (0.10, 1.51)	1.17 (0.28, 4.18)
Unemployed	2.56 (0.04, 3.86)	1.39 (0.30, 3.44)	0.44 (0.05, 2.77)	1.63 (0.10, 3.67)
Educational status				
Illiterate	1.00	1.00	1.00	1.00
Under graduate	0.17 (0.04, 1.11)	0.50 (0.19, 1.97)	0.78 (0.17, 2.76)	0.56 (0.45, 1.68)
College	0.63 (0.04, 4.23)	0.95 (0.18, 2.00)	0.76 (0.10, 1.93)	0.29 (0.05,1.99)
Smoking habit				
yes	1.00	1.00	1.00	1.00
Occasionally	2.72 (0.62, 3.27)	1.07 (0.09, 3.13)	0.69 (0.02,3.34)	0.16 (0.08, 1.62)
No	0.46 (0.07, 1.81)	0.77 (0.19, 1.44)	0.81 (0.30 1.59)	0.56 (0.12, 1.67)
Physical activity				
Inactive	1.00	1.00	1.00	1.00
Minimally active	0.38 (0.29, 6.13)	0.42 (0.15, 0.73)^*^	0.27 (0.09, 0.82) ^*^	0.32 (0.17, 0.84) ^*^
Health enhancing activity	0.91 (0.45, 2.64)	0.50 (0.20, 0.81)^*^	0.49 (0.17, 0.92) ^*^	0.38 (0.16, 0.73) ^*^
Family history of dyslipidemia				
No	1.00	1.00	1.00	1.00
Yes	2.97 (0.54, 16.08)	2.25 (0.67, 7.73)	1.78 (1.30, 2.43) ^***^	1.56 (0.17, 3.14)
Overweight and obesity				
No	1.00		1.00	
Yes	5.07 (1.17, 9.44) ^*^	1.93 (1.07, 3.19) ^*^	10.34 (2.85, 37.24) ^***^	2.14 (1.06, 6.98) ^*^
Conicity index				
No	1.00	1.00	1.00	
Yes	1.81 (0.27, 11.10)	2.6 (0.80, 8.20)	3.04 (1.27, 12.58) ^*^	1.01 (0.34, 2.31)
Generalized Anxiety Disorder				
No anxiety	1.00	1.00	1.00	1.00
Mild	1.83 (0.14, 4.49)	1.34 (0.60, 3.58)	0.79 (0.31, 1.91)	1.35 (0.36, 5.51)
Moderate	1.57 (0.17, 1.73)	1.58 (0.16, 2.29)	1.10 (0.26, 3.13)	1.35 (0.58, 4.96)
Severe	1.72 (0.46, 3.93)	1.61 (0.12, 3.38)	1.27 (1.07, 3.26) ^*^	2.19 (0.31, 6.73)^*^
Having Hypertension				
No	1.00	1.00	1.00	
Yes	1.07 (0.33, 3.66)	0.94 (0.42, 2.27)	1.22 (0.82, 1.93)	1.43 (0.6, 3.85)
Having Diabetes				
No	1.00	1.00	1.00	
Yes	1.34 (0.25, 7.29)	1.05 (0.26, 4.31)	5.57 (1.14, 27.26) ^*^	1.65 (0.16, 2.63)
Salt intake (gr/day)				
Tertile 1	1.00		1.00	1.00
Tertile 2	2.67 (0.66, 10.08)	2.95 (1.14, 7.62) ^*^	1.86 (0.33, 2.14)	1.00 (0.37, 2.20)
Tertile 3	2.16 (0.51, 9.27)	2.80 (1.16, 6.71) ^*^	4.16 (1.44, 11.09) ^**^	1.37 (0.15, 3.29)
Refined carbohydrate intake (gr/day)				
Tertile 1	1.00	1.00	1.00	1.00
Tertile 2	1.16 (0.31, 3.58)	1.37 (0.04, 4.34)	1.97 (1.22, 5.73) ^*^	1.60 (0.55, 3.81)
Tertile 3	1.15 (0.27, 2.59)	1.74 (0.02, 3.12)	1.48 (1.13, 4.16) ^*^	1.28 (0.69, 3.00)
Saturated fat intake (gr/day)				
Tertile 1	1.00	1.00	1.00	1.00
Tertile 2	1.08 (0.09, 1.48)	1.39 (0.01, 4.12)	1.54 (0.07, 2.19)	1.74 (0.39, 4.93)
Tertile 3	1.21 (0.39, 2.16)	1.63 (0.02, 5.38)	1.23 (0.01, 2.55)	1.18 (0.06, 3.29)

* *P*<0.05,

** *P*<0.01,

*** *P*<0.001, Multiple logistic regressions considering the simultaneous effect of all the explanatory variable

According to the results of adjusted logistic regression, the risk of dyslipidemia was higher in overweight and obese men and women. Family history of hyperlipidemia and higher levels of salt intake were associated with high TC levels in men. Moreover, abdominal obesity, anxiety and higher refined carbohydrate intake were associated with high TG levels. Regional residents were more likely to have low HDL-C. Additionally, the risk of high TC and low HDL-C was lower in non-smoker subjects.

In women, being active was a protective factor for high TC, high TG and low HDL-C levels. Higher intake of salt was related to greater risk of high TC levels. In addition, there was an association between high TG levels and abdominal obesity, having diabetes and higher refined carbohydrate intake. Additionally, regional residents were more likely to had low levels of HDL-C. In males and females, age, marriage, employment, education status, and hypertension were not associated with dyslipidemia.

## Discussion

In the present study the prevalence of dyslipidemia and its associated factors in the urban and rural region of East Azerbaijan.

The prevalence of hypercholesterolemia (29.6%) in this study was higher than the reported value in Nepal (17.2%) (8) and India (23.2%) (9) and lower than the values estimated prevalence in U.S (40.5%) (10) and Portugal (56.7%) (11). The prevalence of hyper LDL-cholesterolemia (10.3%) and hypo HDL-cholesterolemia (62%) was respectively lower and higher than the reports from the US (11), Switzerland (12) and Turkey ((13). Hypertriglyceridemia prevalence (41.8%) was lower than the reports from Nepal (8); however, it was higher than the most other western or eastern countries such as US (27%) (11), Italy (19.2%) (14), Portugal (26%) (10) and India (37.7%) (9). This discrepancy between the results from different countries may be due to differences in genetic aspects, obesity prevalence and dissimilarities in lifestyle.

According to the results of present study, the main forms of dyslipidemia were hypo HDL-cholesterolemia and hypertriglyceridemia. These findings were in line with the reports of other Asian countries (15, 16). These observations may be due to the higher intake of simple carbohydrates fat that could affect the level of serum triglyceride (17). In conformity with other studies (18), the prevalence of dyslipidemia was higher in women compared with men. This finding may be related to the higher prevalence of obesity and abdominal obesity in women. Additionally, in this study, the prevalence of obesity (32.2% versus 15.1%) and abdominal obesity (81.4% versus 68.6%) was significantly higher in women compared to men.

Furthermore, in male subjects, a significant increase was observed in the prevalence of LDL-cholesterolemia and hypertriglyceridemia with increasing age. In women, increasing age was related to the prevalence of high TC. In multiple regression analysis, no significant association was found between age and any of the lipid components. This finding may be related to the high prevalence of dyslipidemia in both young and middle-aged adults in this population.

Similar to previous studies (19, 20, 21), obesity was identified as risk factors for all types of dyslipidemia in our study both in men and women. Hence, as a first-stage screening tool, high BMI may be considered to detect dyslipidemic individuals among Iranian adults.

Moreover, family history of dyslipidemia, sedentary lifestyle, smoking habits, salt intake and anxiety were other risk factors for different components of dyslipidemia in men and women.

In addition, in this study, for the first time, high levels of salt intake had positive association with hypercholesterolemia. Adding salt to food can lead to passive overconsumption of fat and calorie in adults. Besides, fat and salt are common and appetitive combinations in food. Therefore, higher salt intake increases the possibility of being overweight and consecutively higher prevalence of elevated TC and dyslipidemia (22).

In accordance with previous study (23), we showed that anxiety was related to higher prevalence of elevated TG. The hypothesis is that activated hypothalamic-pituitary-adrenocortical (HPA) axis in response to stress resulted in increased atherogenic lipid profile.

In this study, being a regional residence increased the prevalence of low HDL level. Rural subjects in Iran tended to have higher animal fat intake which is highly possible cause of higher prevalence of low HDL-C and dyslipidemia in rural areas.

Similar to previous reports, in this study, being active was a protective factor for high TC, high TG and low HDL-C levels in women. Physical activity could effects on blood cholesterol and other lipids by increasing their metabolism. However, its effect of LDL-cholesterol is low and also it has been suggested the cholesterol-lowering effect of dietary interventions is higher than exercise (14). Therefore, it is suggested to merge diet with exercise.(24)

The results of current study showed that approximately 9.2% of the patients with dyslipidemia were aware of their disease and only half of them were receiving lipid-lowering medication or TLC. The awareness in this study was approximately similar to the reported data from China (9.9%) and it was significantly lower than reports from USA (63%). Women were significantly more aware than men. The exact reasons for the observed differences between males and females are unidentified; however, women express their health problem more than men and also seeking healthcare services more than men (25).

## Conclusion

Dyslipidemia, mainly hypertriglyceridemia and low HDL-C, is very common in Azerbaijan, Iran and knowledge and control of dyslipidemia in this population was low. Therefore, implementing proper educational programs to increase health literacy regarding the importance of regular drug consumption, physical activity, anxiety management, proper nutrition, are essential especially among old population. Moreover, programs to improve the surveillance systems and appropriate intervention programs are desired.

## Ethical considerations

Ethical issues (Including plagiarism, informed consent, misconduct, data fabrication and/or falsification, double publication and/or submission, redundancy, etc.) have been completely observed by the authors.
